# Evidence of Energy Metabolism Alterations in Cultured Neonatal Astrocytes Derived from the Ts65Dn Mouse Model of Down Syndrome

**DOI:** 10.3390/brainsci12010083

**Published:** 2022-01-06

**Authors:** Bruna L. Zampieri, Alberto C. S. Costa

**Affiliations:** 1 Division of Pediatric Neurology, Department of Pediatrics, Case Western Reserve University, Cleveland, OH 44106-6090, USA; 2 Hospital Israelita Albert Einstein, São Paulo 05652-900, SP, Brazil; 3 Department of Psychiatry, Case Western Reserve University, Cleveland, OH 44106-6090, USA; 4 Department of Macromolecular Science and Engineering, Case Western Reserve University, Cleveland, OH 44106-6090, USA

**Keywords:** down syndrome, Ts65Dn, astrocytes, mitochondria, oxidative phosphorylation, glycolysis

## Abstract

For many decades, neurons have been the central focus of studies on the mechanisms underlying the neurodevelopmental and neurodegenerative aspects of Down syndrome (DS). Astrocytes, which were once thought to have only a passive role, are now recognized as active participants of a variety of essential physiological processes in the brain. Alterations in their physiological function have, thus, been increasingly acknowledged as likely initiators of or contributors to the pathogenesis of many nervous system disorders and diseases. In this study, we carried out a series of real-time measurements of oxygen consumption rate (OCR) and extracellular acidification rate (ECAR) in hippocampal astrocytes derived from neonatal Ts65Dn and euploid control mice using a Seahorse XFp Flux Analyzer. Our results revealed a significant basal OCR increase in neonatal Ts65Dn astrocytes compared with those from control mice, indicating increased oxidative phosphorylation. ECAR did not differ between the groups. Given the importance of astrocytes in brain metabolic function and the linkage between astrocytic and neuronal energy metabolism, these data provide evidence against a pure “neurocentric” vision of DS pathophysiology and support further investigations on the potential contribution of disturbances in astrocytic energy metabolism to cognitive deficits and neurodegeneration associated with DS.

## 1. Introduction

Down syndrome (DS), the genetic disorder typically resulting from the triplication of human chromosome 21, is the most prevalent genetically defined cause of intellectual disability [1,2]. In addition to a significant reduction in mean development/intellectual quotient (DQ/IQ), individuals with DS present a disproportionate impairment in certain cognitive areas such as short-term and episodic memory, expressive language, and executive function [3,4,5]. For many decades, neurons have been the central focus of studies aiming to better understand the mechanisms underlying the cognitive impairment and the neurodevelopmental and neurodegenerative aspects of DS. In recent years, the recognition that other brain cell types, especially astrocytes, have crucial roles in the maintenance of proper brain development and function, including neuronal integrity, has opened new avenues in neuroscience and DS research [6,7].

Astrocytes, once thought to have only a passive role, are now acknowledged as active participants of a variety of essential physiological processes in the brain. These cells control the extracellular homeostasis of ions and water, as well as axon and dendrite outgrowth, participate in the formation, maturation, and modulation of synapses, and regulate the clearance of neurotransmitters such as glutamate [8,9,10]. Indeed, one of the most important functions of astrocytes is neurotransmitter removal from the synaptic cleft. In the case of glutamate, astrocytes take up extracellular glutamate by recruiting glutamate transporters and mitochondria to perisynaptic processes [11,12]. Astrocytic glutamate is converted into glutamine, which is released into the extracellular space and taken up by neurons as the material to produce glutamate in what is known as the glutamate–glutamine cycle [13]. The glutamate–glutamine cycle between neurons and astrocytes becomes essential as it protects neurons against glutamate excitotoxicity [14]. Astrocytes also play an important role in providing energy substrates such as pyruvate and lactate to neurons to assist with their high-energy demands particularly during neuronal activation. Glutamate released from active neurons activates astrocytic glycolysis, leading to production of lactate, which is subsequently shuttled to neurons as energy fuel [15]. This astrocytic–neuronal metabolic coupling is thought to play a central role in long-term neuronal plasticity and memory formation [16,17,18]. Therefore, alterations in astrocyte physiological functions have been increasingly recognized as likely initiators of or contributors to the pathogenesis of many nervous system disorders and diseases [6]. Considering the role of mitochondrial metabolism in proper astrocyte functioning [19], disturbances in astrocytic energy metabolism can potentially result in dysfunctional neuronal activity and ultimately in cognitive impairment.

Mitochondrial dysfunction has long been associated with DS. Studies in diverse tissues or cell types and at different ages, from both human subjects and animal models, have reported alterations that include aberrant mitochondrial structure, function, and energy metabolism with an impairment of reactive oxygen species (ROS) homeostasis [20,21]. Many genes and miRNAs located in chromosome 21 are known to be involved in mitochondrial pathways [21,22,23,24] and, therefore, might play a role in the mitochondrial impairment observed in DS. Interestingly, a recent review hypothesized that DS is an oxidative phosphorylation disorder [25], and another recent study proposed that the dysregulation within mitochondrial oxidative phosphorylation complexes is an early marker of cognitive decline in DS [26].

In light of our current understanding of the importance of astrocytes to brain function and the hypothesized role of mitochondrial dysfunction in the pathogenesis of DS, we set out to expand the available knowledge on astrocyte energy metabolism in this chromosomal disorder. To this end, we produced primary astrocyte cultures from neonatal Ts65Dn mice, which are the best characterized mouse model of DS [27]. We then assessed mitochondrial functioning and cellular energy profiles in these Ts65Dn-derived astrocyte cultures by simultaneously measuring mitochondrial respiration (oxygen consumption) and glycolysis (extracellular acidification) and compared these measurements with those obtained from primary astrocyte cultures of age-matched wild-type control (WT) mice.

## 2. Materials and Methods

### 2.1. Animals

Day 0 and 1 (P0/P1) neonatal Ts65Dn and WT mice were used in the study. P0/P1 were simply the earliest postnatal ages we could select (note that, if we selected an embryonic age, that would have meant sacrificing the Ts65Dn mothers, which is particularly consequential for colony viability, because females are the only viable Ts65Dn breeders in this mouse model of DS). Ts65Dn mice were generated by repeated backcrossing of Ts65Dn females with B6EiC3Sn.BLiAF1/J hybrid male mice (C57BL/6JEiJ females crossed to C3Sn.BLiA-Pde6b^+^/Dn males) in the Animal Resource Center (ARC) at Case Western Reserve University (CWRU). Mice were genotyped by polymerase chain reaction (PCR) as described by Duchon et al. [28]. WT mice for this study were the littermate euploid mice identified in this PCR genotyping process. All mice were housed under a 12 h light/dark cycle and had ad libitum access to food and water. All experimental procedures and protocols strictly conformed to the Guide for the Care and Use of Laboratory Animals (National Institutes of Health) and were approved by the Institutional Animal Care and Use Committee (IACUC) at CWRU.

### 2.2. Primary Cultures

Single-cell suspensions from neonatal mice hippocampus were prepared by enzymatic dissociation with 0.25% *w*/*v* trypsin-EDTA in Hanks’ balanced salt solution (HBSS). In order to isolate neurons and non-neuronal cells we performed magnetic-activated cell sorting (MACS) using the Neuron Isolation Kit, mouse (Miltenyi Biotec Inc., Auburn, CA, USA), according to the manufacturer’s instructions. Neuronal cells were isolated by depletion of non-neuronal cells and used elsewhere. Highly enriched non-neuronal cells were indirectly magnetically labeled using biotin-conjugated antibodies specific for non-neuronal cells in combination with Anti-Biotin MicroBeads. Non-neuronal cells suspensions were plated onto uncoated dishes at a 2.5 × 10^4^ cells/cm^2^ density and cultured in astrocyte medium containing high glucose (4.5 g/L) Dulbecco’s modified Eagle medium (DMEM) supplemented with 10% fetal bovine serum, N-2 Supplement (100×), 100 units/mL penicillin, and 100 μg/mL streptomycin, and epidermal growth factor (20 ng/mL) to enhance astrocyte proliferation. During the first week, media were changed every other day until cells were confluent (around day 7, DIV7). From the second week on, once astrocytes cultures reached confluence, they were trypsinized and replated onto six-well plates at 2.5 × 10^4^ cells/cm^2^ density in astrocyte medium containing 0.25 mM dBcAMP to induce differentiation. Every 3–4 days, the medium was replaced, and the cultures were used for experiments on DIV 14–21 (cells were detached with trypsin and replated at least twice before use). Purity of the primary astrocyte culture was assessed by GFAP (an astrocyte-specific intermediate filament) staining, and CD11b was used as a microglial marker.

### 2.3. Metabolic Flux Analyses

Oxygen consumption rate (OCR) and extracellular acidification rate (ECAR) were measured using a Seahorse XFp Flux Analyzer (Agilent Technologies, Santa Clara, CA, USA) with a Seahorse XF Cell Mito Stress Test Kit (Agilent Technologies, Santa Clara, CA, USA) following the manufacturer’s instructions. OCR was calculated as a measure of aerobic respiration, and ECAR was calculated as a measure of lactic acid production in anaerobic glycolysis. Briefly, cells were seeded on a microplate (Agilent Technologies, Santa Clara, CA, USA) in triplicate at predetermined cell densities (20,000–30,000 cells/well) and incubated overnight at 37 °C and 5% CO_2_ before experiments. Two background wells without cells were included in all assays. Prior to starting the assay, cells were washed and incubated in Seahorse Assay Medium supplemented with 25 mM glucose, 4 mM glutamine, and 1 mM sodium pyruvate (pH 7.4) in a 37 °C incubator without CO_2_ for 45 min. OCR (pMoles O_2_/min) and ECAR (mpH/min) were measured under basal conditions followed by the sequential addition of 4 μM oligomycin, 2 μM carbonyl cyanide-4-(trifluoromethoxy) phenylhydrazone (FCCP), and 1 μM rotenone/antimycin A. All respiratory modulators were used at ideal doses established by preliminary titrations. The sequential compound injections measure basal respiration, ATP production, proton leak, maximal respiration, spare respiratory capacity, and nonmitochondrial respiration. In order to investigate mitochondrial respiration during glutamate uptake, glutamate was injected at a final concentration of 200 μM before oligomycin, FCCP, and rotenone/antimycin A in a group of experiments. Data were collected and extracted using the Wave software 2.6.1 (Agilent Technologies, Santa Clara, CA, USA). Values were normalized to total astrocyte numbers according to cell-permeant nuclear counterstain with NucBlue^®^ Live ReadyProbes^®^ Reagent (Invitrogen, Carlsbad, CA, USA). The numbers of immunostained astrocytes in the culture dishes were quantified using images taken by the EVOS FL Auto 2 Cell Imaging System (Invitrogen, Carlsbad, CA, USA) at 20× magnification. The number of immunostained astrocytes in 18 nonoverlapping microscopic fields taken from each well was counted using Igor Pro 8. Finally, the total number of stained cells in each well was estimated by extrapolating the counts for the entire well area (0.106 cm^2^).

### 2.4. H_2_O_2_ Treatment

To assess the response of astrocytes to oxidative stress, cells were stimulated with H_2_O_2_, and cytotoxicity was measured using the colorimetric 3-(4,5-dimethylthiazol-2-yl)-2,5-diphenyltetrazolium bromide (MTT) reduction assay. Briefly, 3 × 10^4^ astrocyte cells were seeded onto 48-well plates. Forty-eight hours later, media were changed to serum-free medium with various concentrations of H_2_O_2_ (0–1050 µM). After 24 h, the cells were incubated with MTT (5 mg/mL) in phosphate-buffered saline (PBS) working solution at 37 °C for another 4 h. The formazan crystals were dissolved in dimethyl sulfoxide, and the absorbance was read at 540 nm by a microplate reader (Tecan Infinite M1000). Cell viability was expressed as a percentage of untreated cells. All MTT assays involved between six and 12 samples for each group and condition, which were measured in triplicate. Values were normalized to the total protein per well after the completion of the assay by the Bradford protein assay.

### 2.5. Immunocytochemistry

Cells were fixed with 4% paraformaldehyde in PBS for 30 min, at room temperature. The membranes of fixed cells were permeabilized by treatment with 0.3% Triton X-100 made up in PBS containing 5% normal goat serum (blocking solution) at room temperature, for 1 h. Next, they were incubated with primary antibodies: mouse-anti-GFAP (1:800, Novus Biologicals, Centennial, CO, USA) and rat-anti-CD11b (1:500, Novus Biologicals, Centennial, CO, USA) in PBS with 1% goat serum and 0.1% Triton X-100 at 4 °C overnight. After three 10 min washes in PBS, cells were incubated with secondary antibodies to goat anti-mouse IgG (1:250, Thermo Fisher Scientific, Waltham, MA, USA) or goat anti-rat IgG (1:500, Thermo Fisher Scientific, Waltham, MA, USA) for 1.5 h at room temperature. Nuclei were counterstained with 4′,6-diamidino-2-phenylindole (DAPI, Thermo Fisher Scientific, Waltham, MA, USA) for 10 min at room temperature. Immunofluorescence was visualized with the EVOS FL Auto 2 Cell Imaging System (Invitrogen, Carlsbad, CA, USA).

### 2.6. Statistical Analysis

Data were analyzed using Seahorse Wave Software (version 2.6.1., Agilent Technologies, Santa Clara, CA, USA), Statistica Academic (version 13, TIBCO Sofware, Palo Alto, CA, USA), and GraphPad Prism (version 7.0, GraphPad Sofware, La Jolla, CA, USA). Data are reported as the mean ± SEM, and the number of experiments is indicated in each case. Multiple comparisons (using Statistica) were performed by one-way analysis of variance (ANOVA), followed by Fisher LSD post hoc tests, or by repeated-measures ANOVA (RM-ANOVA), also followed by Fisher LSD tests when genotype dependence was detected. Pairwise comparisons (using GraphPad Prism) were conducted by unpaired two-tailed Student’s *t-*tests with Welch’s correction for normally distributed data and Mann–Whitney U test when normal distribution could not be verified. Differences were considered significant when *p*-values were <0.05. (Raw data and Output from statistical software packages can be found in Supplementary Materials).

## 3. Results

**Figure 1:**
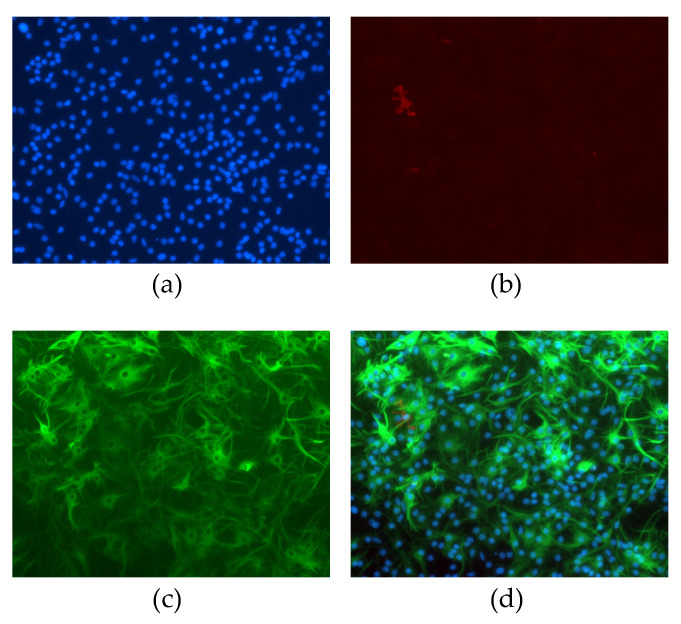
Non-neuronal neonatal cells after magnetic isolation and 15 days cultured in vitro. Cells were stained with (**a**) DAPI (blue), (**b**) CD11b (red), or (**c**) GFAP (green). Panel (**d**) represents merged images.

Astrocyte cultures had purity greater than 99% as identified by immunofluorescence staining against GFAP (an astrocyte-specific intermediate filament) and CD11b (a marker for microglia) (Figure 1).

### 3.1. Basal Conditions

**Figure 2:**
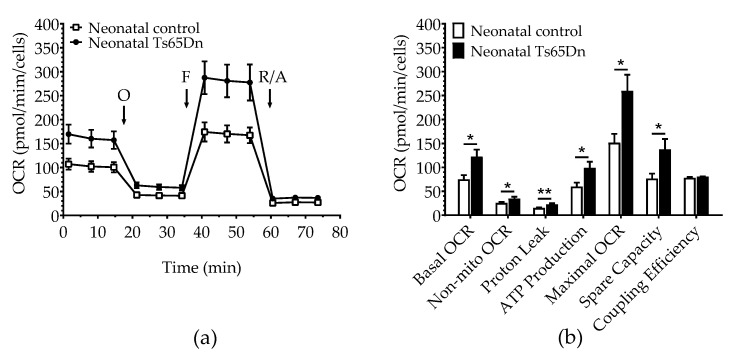
Oxygen consumption in astrocytes derived from neonatal Ts65Dn and control mice. (**a**) Real-time changes in oxygen consumption rate (OCR) after treatment with oligomycin (O), FCCP (F), and rotenone/antimycin A (R/A) (**b**) Individual parameters derived from OCR profiles. Basal OCR, nonmitochondrial OCR (Non-Mito), proton leak, ATP-linked OCR, maximal OCR, and spare capacity were significantly increased in astrocytes derived from Ts65Dn neonatal mice compared with those from neonatal control mice. Data are expressed as the mean ± SEM (*n* = 10–13 independent experiments with samples in triplicate); * *p* < 0.05, ** *p* < 0.01.

We used the Seahorse Extracellular Flux Analyzer to compare astrocyte bioenergetics of neonatal Ts65Dn and WT cultured cells. Basal OCR (Student’s *t-*test, t = 2.823; df = 18.93; *p* = 0.011), non-mitochondrial OCR (Mann–Whitney, U = 33.0, *p* = 0.049), proton leak (Student’s *t-*test, t = 3.257; df = 15.95; *p* = 0.005), ATP production (Mann–Whitney U = 25.0, *p* = 0.012), maximal OCR (Student’s *t-*test, t = 2.819; df = 18.03; *p* = 0.011), and spare OCR (Student’s *t-*test, t = 2.567; df = 16.86; *p* = 0.020) were significantly higher in neonatal Ts65Dn astrocytes compared with neonatal WT astrocytes. Coupling efficiency was not significantly different between genotypes (Student’s *t-*test, t = 1.231; df = 11.88; *p* = 0.242) (Figure 2). Neonatal Ts65Dn astrocytes showed a 1.6-fold increase in basal oxygen consumption compared with neonatal WT astrocytes. However, under conditions in which basal respiration is significantly different between the experimental groups, it is important to consider the mitochondrial measurements as percentages of basal respiration. When we compared the measurements as a percentage of basal OCR for neonatal Ts65Dn and WT mice, non-mitochondrial OCR was still significantly higher in neonatal Ts65Dn astrocytes compared with WT ones (Student’s *t-*test, t = 3.203; df = 14.36; *p* = 0.006). However, maximal OCR (Student’s *t-*test, t = 0.886; df = 17.32; *p* = 0.388), proton leak (Student’s *t-*test, t = 1.308; df = 11.67; *p* = 0.216), ATP production (Student’s *t-*test, t = 0.975, df = 13.1; *p* = 0.347), and spare OCR (Student’s *t-*test, t = 1.013; df = 18.05, *p* = 0.324) were no longer different between the groups.

**Figure 3:**
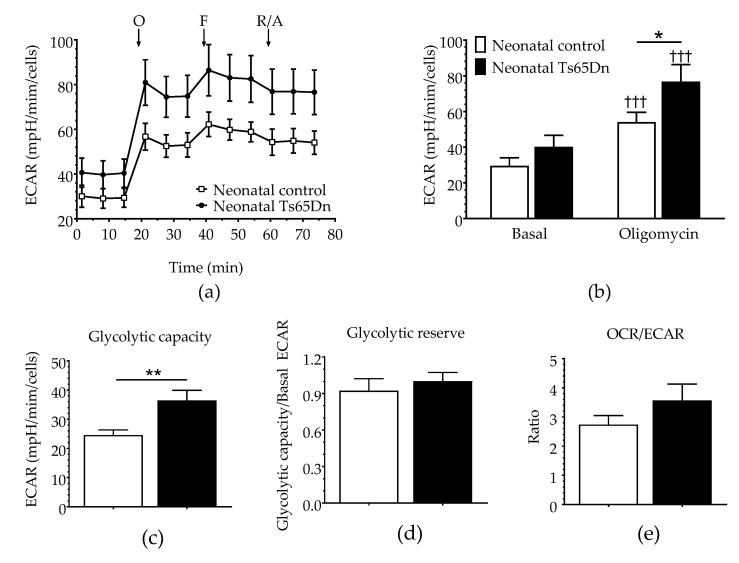
Glycolysis in astrocytes derived from neonatal Ts65Dn and control mice. (**a**) Real-time changes in extracellular acidification rate (ECAR) after treatment with oligomycin (O), FCCP (F), and rotenone/antimycin A (R/A). (**b**) Basal and oligomycin ECAR (ECAR after oligomycin treatment) derived from ECAR profiles. (**c**) Glycolytic capacity (calculated by subtracting the basal ECAR value from the ECAR value after oligomycin treatment). (**d**) Glycolytic reserve (calculated by dividing the glycolytic capacity by the basal ECAR value). (**e**) OCR/ECAR ratio. Data are expressed as the mean ± SEM (*n* = 10–13 independent experiments with samples in triplicate); * *p* < 0.05, ** *p* < 0.01, and ††† *p* < 0.001 indicate significant differences between before and after treatment for a given genotype.

Glycolytic parameters were assessed simultaneously during the same assay using the ECAR before (basal ECAR) and after the cells were stressed by oligomycin, which can effectively shut down oxidative phosphorylation (Figure 3). Although RM-ANOVA did not show a significant genotype dependence (F_(1,21)_ = 2.751, *p* = 0.112), it detected both a significant treatment dependence (F_(1,21)_ = 210.305, *p* < 0.001) and interaction between genotype and treatment (F_(3,21)_ = 7.976, *p*= 0.010), which led us to perform post hoc tests (Fisher LSD) to differentiate between the means of each individual assessment. Although significant before vs. after treatment differences were observed for both genotypes (three daggers on top of the oligomycin bars indicating *p* < 0.001), no significant differences in basal ECAR were detected between WT and Ts65Dn astrocytes (*p* = 0.307). However, after treatment with oligomycin (Oligo ECAR, indicative of glycolytic activity), neonatal Ts65Dn astrocytes presented statistically significant higher ECAR compared with neonatal WT astrocytes (*p* = 0.038). Additionally, Ts65Dn astrocytes also showed a significantly increased glycolytic capacity (calculated by subtracting the basal ECAR from the oligomycin-induced ECAR) when compared with those from neonatal WT (Mann–Whitney, U = 17, *p* = 0.002). Glycolytic reserve, which is equal to glycolytic capacity divided by basal ECAR, did not differ between astrocytes derived from neonatal Ts65Dn and WT mice (Student’s *t-*test, t = 0.523; df = 17.23; *p* = 0.653). The OCR/ECAR ratio was also determined to assess the relative contribution of mitochondrial respiration versus glycolysis to energy generation (Figure 3). The OCR/ECAR ratios in astrocytes derived from neonatal WT and Ts65Dn mice were also not significantly different from each other (Mann–Whitney U = 46.0, *p* = 0.257).

### 3.2. Glutamate Stimulation

**Figure 4:**
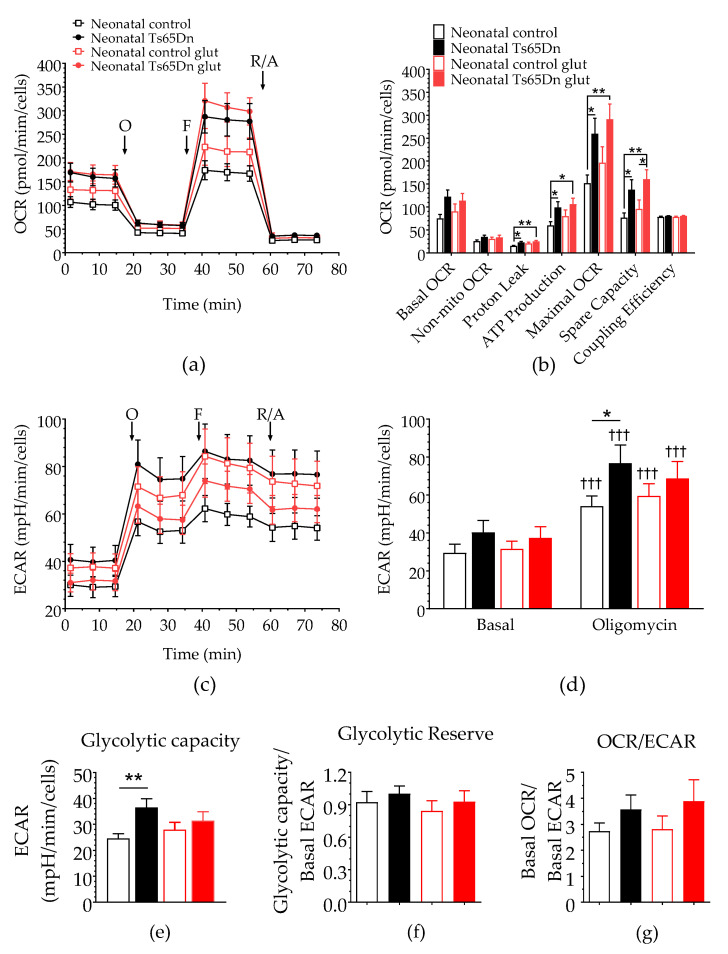
Effect of glutamate stimulation on astrocytic bioenergy in neonatal Ts65Dn and control mice. (**a**) Real-time changes in oxygen consumption rate (OCR) after treatment with oligomycin (O), FCCP (F), and rotenone/antimycin A (R/A). (**b**) Individual parameters derived from OCR profiles. Spare respiratory capacity was significantly higher in neonatal Ts65Dn astrocytes compared with neonatal control astrocytes after addition of glutamate. (**c**) Real-time changes in extracellular acidification rate (ECAR) after treatment with O, F, and R/A. (**d**) Basal and oligomycin ECAR (ECAR after oligomycin treatment) derived from ECAR profiles. (**e**) Glycolytic capacity (calculated by subtracting the basal ECAR value from the ECAR value after oligomycin treatment), which did not differ between the groups after glutamate injection. (**f**) Glycolytic reserve (calculated by dividing the glycolytic capacity by the basal ECAR value and (**g**) OCR/ECAR ratio. Data are expressed as the mean ± SEM (*n* = 9–13 independent experiments with samples in triplicate); * *p* < 0.05, ** *p* < 0.01, and ††† *p* < 0.001 indicate differences between before and after treatment for a given genotype.

To further assess astrocyte bioenergetics, both OCR and ECAR of astrocytes derived from neonatal Ts65Dn and WT mice were measured after 200 μM glutamate stimulation (Figure 4). One-way ANOVA did not detect any significant difference in basal OCR (F_(3,37)_ = 2.485, *p* = 0.076), nonmitochondrial OCR (F_(3,37)_ = 1.210, *p* = 0.320), or coupling efficiency (F_(3,37)_ = 1.100, *p* = 0.360) between neonatal Ts65Dn and WT astrocytes caused by the presence of glutamate. However, we detected significant genotype–treatment effects on proton leak (F_(1,37)_ = 3.718, *p* = 0.020), ATP production (F_(3,37)_ = 2.891, *p* = 0.048), maximal OCR (F_(3,37)_ = 3.851, *p* = 0.017), and spare capacity (F_(3,37)_ = 3.783, *p* = 0.018). Post hoc analysis showed an interesting pattern in which mean values for measures that were significantly higher in Ts65Dn astrocytes than those in WT astrocytes at baseline were no longer statistically different between genotypes after glutamate stimulation. This was true for proton leak (*p* = 0.011 and *p* = 0.197 before and after glutamate stimulation, respectively), ATP production (*p* = 0.021 and *p* = 0.164), and maximal OCR (*p* = 0.015 and *p* = 0.052). Spare respiratory capacity, however, remained significantly higher for Ts65Dn astrocytes when compared with WT astrocytes even after glutamate treatment (*p* = 0.024 and *p* = 0.031 before and after glutamate stimulation, respectively). This difference was maintained when spare respiratory capacity was analyzed as a percentage of basal OCR.

RM-ANOVA of the glycolysis parameters included treatment (basal and oligomycin) as the categorical factors and the combination of genotype and glutamate treatment (WT no-glutamate, WT glutamate, Ts65Dn no-glutamate, and Ts65Dn glutamate) as the dependent variables. Similar to the baseline analysis shown in Figure 2b, this analysis did not show a significant dependence on the categorical factors (F_(3,37)_ = 1.282, *p* = 0.295). However, we detected both a significant treatment dependence (F_(1,37)_ = 376.868, *p* < 0.001) and an interaction between categorical factors and treatment (F_(3,37)_ = 3.043, *p* = 0.041), which led us to perform post hoc tests (Fisher LSD) on the data. Although significant before vs. after treatment differences were observed for both genotypes (three daggers on top of the oligomycin bars indicating *p* < 0.001), no significant differences in basal ECAR were detected between WT and Ts65Dn astrocytes with (*p* = 0.264) or without (*p* = 0.591) glutamate treatment. Once again, a significant difference between WT and Ts65Dn astrocytes revealed by oligomycin treatment (*p* = 0.022) was ‘obscured’ by glutamate treatment (*p* = 0.394). The one-way ANOVA glycolytic capacity of astrocytes treated with glutamate found a significant genotype–treatment effect (F_(3,37)_ =  3.043, *p* = 0.041). As revealed by post hoc analysis, this effect was restricted to a significant difference between WT and Ts65Dn astrocytes under basal conditions (*p* = 0.007), which was no longer significant after glutamate treatment (*p* = 0.465). Lastly, similar analysis showed no significant genotype–treatment effect on glycolytic reserve (F_(3,37)_ =  0.555, *p* = 0.648) or OCR/ECAR ratio (F_(3,37)_ = 0.924, *p* = 0.439).

### 3.3. MTT Assay

**Figure 5:**
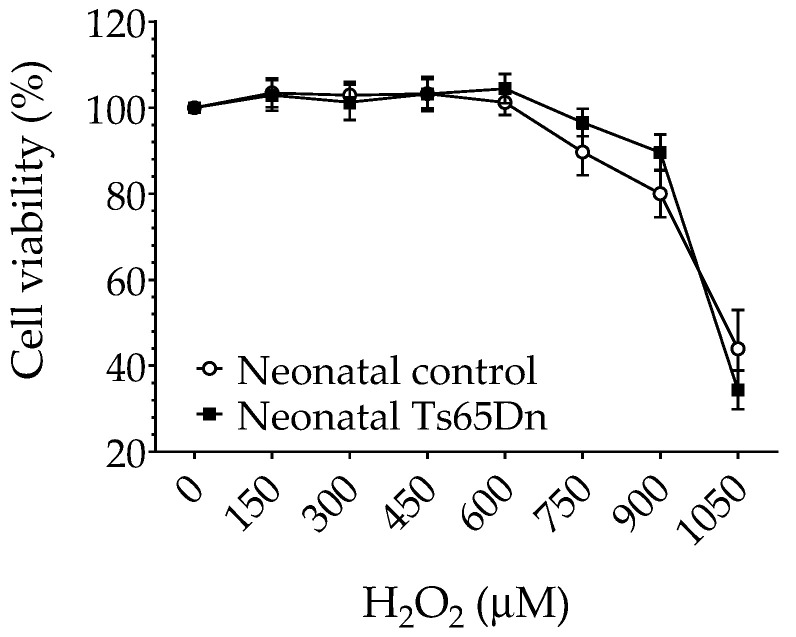
Cytotoxicity of hydrogen peroxide in neonatal control and Ts65Dn astrocytes measured by MTT reduction expressed as a percentage of untreated control cells. Cultures were exposed to the indicated concentrations of hydrogen peroxide added to the culture media for 24 h. Data are the means ± SEM, *n* = 6–12/group performed in triplicate.

To determine whether neonatal Ts65Dn astrocytes alter their susceptibility to oxidative stress in comparison with neonatal WT astrocytes, we investigated the effect of H_2_O_2_ exposure on astrocyte viability using the MTT assay. No differences were found in cell viability between neonatal WT and Ts65Dn astrocytes under our experimental conditions (Figure 5). Although RM-ANOVA showed a significant H_2_O_2_ dose dependence (F_(6,66)_ = 35.196, *p* < 0.0001), no significant genotype dependence (F_(1,11)_ = 0.142, *p* = 0.71) or interaction between H_2_O_2_ concentration and genotype (F_(6,66)_ = 0.842, *p* = 0.5418) was detected.

## 4. Discussion

In this study, we carried out a series of real-time measurements of OCR and ECAR, indicators of OXPHOS activity and glycolysis, respectively, in hippocampal astrocytes from neonatal Ts65Dn and WT mice. Our results revealed a significant increase in basal OCR in neonatal Ts65Dn astrocytes compared with astrocytes derived from neonatal WT mice, indicating an increased OXPHOS metabolism for hippocampal astrocytes.

OCR measures the flux of electrons through the respiratory chain, as well as all the processes in the cell capable of consuming energy [29]. The heightened astrocytic mitochondrial function observed in neonatal Ts65Dn mice does not seem to be a generalized energetic phenotype, given that glycolysis (i.e., ECAR) was not significantly higher in neonatal Ts65Dn astrocytes compared with neonatal WT astrocytes. ECAR was only significantly different between these groups after oligomycin injection, which inhibits OXPHOS. In response to inhibition of mitochondrial OXPHOS, neonatal Ts65Dn astrocytes displayed a higher glycolytic capacity than WT-derived astrocytes. However, there was no change in the glycolytic reserve (which indicates the capability of a cell to respond to an energetic demand and may be a useful indicator of cell resilience in times of emergency).

We speculate that the increased OXPHOS activity in neonatal Ts65Dn astrocytes reflects a shift from a more glycolytic to a more oxidative metabolism. Under typical conditions, astrocytes are predominantly glycolytic. They rely on glycolysis to generate ATP and lactate, which is released into the extracellular space and taken up by neurons [15]. Considering the intimate coupling of astrocytic/neuronal bioenergetics, a shift in astrocytes away from anaerobic respiration might have detrimental effects on the brain. The rationale is that astrocytes with increased mitochondrial metabolism utilize energy substrates for their own metabolism rather than transferring them to neurons, which would cause them to neglect their neuro-supportive roles and even potentially gain toxic function. Increased mitochondrial metabolism has been reported in aged, activated, and immune-stimulated astroglia [30]. Jiang and Cadenas [31] demonstrated an age-dependent increase in H_2_O_2_ generation by astrocytes and age-dependent metabolic shift from anaerobic metabolism toward increased mitochondrial metabolism, as well as a switch in astrocytes’ functionality from neurotrophic to neurotoxic. Natarajaseenivasan et al. [32] showed that activation of astrocytes by the viral protein HIV Tat and/or cocaine induced a metabolic switch from glucose to fatty-acid oxidation, leading to enhanced mitochondrial respiration and reduced lactate production, potentially contributing to HIV-associated neurocognitive disorder. A study using functional astrocytes derived from induced pluripotent stem cells (iPSCs) from patients with Alzheimer’s disease (AD) and the PSEN1 ΔE9 mutation, a condition linked to an increase in the accumulation of Aβ amyloid, showed a higher basal OCR compared with isogenic control cells, as well as increased reactive oxygen species and reduced lactate production [33]. When cocultured with healthy neurons, these AD-derived astrocytes contribute to reduced neuronal activity most probably leading to dysfunctional neurons.

When we conducted experiments with neonatal astrocytes during glutamate uptake, basal OCR values did not differ significantly between the groups. However, there was a significant increase in spare respiratory capacity in neonatal Ts65Dn astrocytes compared with neonatal WT-derived astrocytes in response to short-term glutamate application. Spare respiratory capacity, calculated as the difference between the basal and maximal OCR, is a measure of the ability of the cell to respond to increased energy demand or stress [29], which can be an indicator of cell fitness or ability to respond to stress.

In this study, when exposed to the effect of H_2_O_2_, a major form of ROS, both neonatal Ts65Dn and WT astrocytes showed similar cell viability in the different concentrations of H_2_O_2_ (150–1050 μM). These results are different than published findings in primary astrocytes established from human fetal cerebral cortical tissue [34] in which trisomic astrocytes showed greater resistance to H_2_O_2_ than disomic astrocytes in a comparable range of concentrations. However, it is difficult to directly compare results from human and animal brains. There are, for instance, well-known issues related to variability in the quality of tissue samples collected from therapeutic abortions (used as the source of human fetal brains) and to the exact correspondence between fetal and postnatal developmental stages in both species. Furthermore, when studying the cytotoxicity of hydrogen peroxide in neonatal control and Ts65Dn astrocytes, we used the reduction in MTT as the only metric of cell viability. This represents an experimental limitation of this study, given that the reduction in MTT is just a reflection of the cellular dehydrogenase activity, which might not strictly reflect changes in cell viability but might also reflect changes in mitochondrial dehydrogenase activity. In future studies, additional methods to measure viability, such as Trypan blue or Hoechst 33342, should be used to confirm the hydrogen peroxide cytotoxicity findings reported here.

At a first glance, the enhanced mitochondrial metabolism observed in our study may seem to contradict a body of evidence indicating reduced energy metabolism in DS. However, most of the previous studies focused on cell types other than astrocytes or failed to distinguish between neurons and astrocytes. Decreases in oxygen consumption have been reported in human fibroblasts [35,36,37,38,39,40], mouse neural progenitor cells isolated from the hippocampus [41], mouse brain homogenates [42], and human lymphocytes [43]. In contrast, in a recent study, Anderson et al. [35] demonstrated that, under basal conditions, OCR and ECAR, determined using the Seahorse extracellular flux analyzer, are not different in dermal fibroblasts obtained from individuals with and without DS. However, in a different study, mitochondria in trisomy 21 iPSCs were shown to be larger and more metabolically active with a higher rate of O_2_ consumption than isogenic, disomic controls [24]. Therefore, our study underscores the importance of investigating mitochondrial function regionally, as different brain regions and different cell types may present different metabolic profiles. Even different cells within the same tissue such as the brain have unique features of mitochondrial morphology and function with differential enrichment of specific proteins [44].

A potentially fruitful avenue for future studies of bioenergetics alterations in astrocytes would involve the investigation of associations with dysfunctional adult neurogenesis in DS. Astrocytes have been shown to regulate neural progenitor proliferation and differentiation into neuronal lineage, and increasing evidence emphasizes the functional importance of new neuron connectivity within the hippocampal circuitry [45,46]. Moreover, deficits in adult neurogenesis in Ts65Dn mice, which were first observed by Clark et al. [47] well over a decade ago, have now been confirmed by several other research teams [48,49]. Therefore, it would be important to investigate whether the phenotypes we observed in Ts65Dn neonatal astrocytes are also present in adult animals, and whether strategies that rescue adult neurogenesis deficits in Ts65Dn mice would also normalize bioenergetics parameters in astrocytes. Although we performed preliminary measurements of OCR and ECAR in hippocampal astrocytes derived from adult Ts65Dn and euploid control mice (data not shown), these data were inconclusive because we had to pool astrocytes from several animals due to the significantly lower cell numbers obtained from adult mice. This meant that, in our preliminary experiments, the purity of the primary astrocyte culture could not be assessed individually and reliably by GFAP and CD11b. Therefore, future studies using higher resolution (potentially single-cell) methods to identify astrocytes in primary culture should lead to reliable data on this important issue.

The present study represents one of the most complete assessments of mitochondrial bioenergetics in astrocytes of the Ts65Dn mouse model for DS to date. It provides important information regarding the metabolism of these cells at a sensitive stage of neurodevelopment. Any disruption in energy homeostasis, even very small ones, in such a significant period may have negative consequences for healthy neurodevelopment. In view of the importance of astrocytes in metabolic function and the metabolic linkage between astrocytes and neurons, one could speculate that an increase in mitochondrial function in astrocytes may precede the decreased neuronal mitochondrial metabolism that has previously been associated with DS.

## 5. Conclusions

The present study, which examined measurements performed with a Seahorse XFp Flux Analyzer, demonstrates a significant increase in basal oxygen consumption rate in primary cultures of neonatal astrocytes obtained from the Ts65Dn mouse model of Down syndrome when compared with astrocytes derived from neonatal wild-type mice. This indicates a comparatively increased oxidative phosphorylation for Ts65Dn-mouse-derived hippocampal astrocytes. These findings may have important consequences for the understanding of the pathophysiology of the cognitive deficits and neurodegenerative processes associated with Down syndrome.

## Data Availability

All other data will be made available upon request.

## Supplementary Materials

The following supporting information can be downloaded at https://www.mdpi.com/article/10.3390/brainsci12010083/s1, Raw Data and Output from Statistical Software.
