# Supplementary material for: Evidence of Energy Metabolism Alterations in Cultured Neonatal Astrocytes Derived from the Ts65Dn Mouse Model of Down Syndrome
Source: Brain Sci. 2022 Jan 6;12(1):83. doi: 10.3390/brainsci12010083 (PMC8773919; doi:10.3390/brainsci12010083)
Supplement: Supplementary file 1 [file brainsci-12-00083-s001.zip › brainsci-1520392-supplementary.pdf]

Evidence of energy metabolism alterations in cultured neonatal astrocytes derived from the Ts65Dn mouse model of Down syndrome

Bruna L. Zampieri and Alberto C. S. Costa

Supplementary Materials

Raw Data and Output from Statistical Software

Data in Figure 2b (Data Analysis done with GraphPad Prism)

Basal OCR		Non-mito OCR		Proton Leak		ATP Production		Maximal OCR		Spare Capacity		Coupling Efficiency	
WT	Ts65Dn	WT	Ts65Dn	WT	Ts65Dn	WT	Ts65Dn	WT	Ts65Dn	WT	Ts65Dn	WT	Ts65Dn
115.93	213.94	31.2	57.5	19.44	32.89	96.49	181.05	258.46	385.1	142.54	171.16	83.23	84.64
63.35	197.29	21.91	59.83	10.65	34.2	52.7	163.09	135.76	511.76	72.41	314.46	83.18	82.56
60.47	210.49	20.83	53.11	13.75	37.86	46.72	172.63	126.78	454.36	66.31	243.87	77.24	82.01
74.17	82.18	22.89	30.35	17.47	15.37	56.7	66.81	147.8	212.95	73.63	130.77	76.33	81.3
62.38	120.57	22.32	35.25	14.27	24.09	48.11	96.48	128.75	304.73	66.37	184.16	77.12	80.04
132.24	99.67	44.25	28.91	14.83	23.75	117.41	75.92	250.09	245.71	117.84	146.04	88.82	76.17
74.02	95.71	24.56	28.39	16.83	19.48	56.05	76.23	132.37	241.06	59.49	145.35	76.9	79.64
54.65	133.44	23.51	33.72	17.24	21.14	37.99	112.3	99.1	229.57	43.87	96.13	68.77	84.15
47.07	111.45	21.33	29.09	11.1	19.47	36.7	91.99	84.63	199.91	36.83	88.46	76.93	82.39
65.55	101.19	24.13	27.68	16.78	21.71	47.97	76.96	152.11	128.88	87.37	30.21	74.1	77.99
	73.33		21.19		14.54		56.78		165.28		93.95		79.62
	60.14		20.69		13.24		45.96		139.42		80.22		77.67
	92.79		28.11		18.83		72.98		157.49		65.68		79.49

Basal OCR	WT		Ts65Dn
D'Agostino & Pearson normality test			
K2	5.596		2.514
P value	0.0609		0.2845
Passed normality test (alpha=0.05)?	Yes		Yes
Table Analyzed		Basal OCR	
Column B		Ts65Dn postnatal	
vs.		vs.	
Column A		WT postnatal	
Unpaired t test with Welch's correction			
P value		0.0109	
P value summary		*	
Significantly different (P < 0.05)?		Yes	
One- or two-tailed P value?		Two-tailed	
Welch-corrected t, df		t=2.823 df=18.93	

Non-mito OCR	WT		Ts65Dn
D'Agostino & Pearson normality test			
K2	17.56		3.307
P value	0.0002		0.1914
Passed normality test (alpha=0.05)?	No		Yes
P value summary	***		ns
Table Analyzed		Non-mitochondrial Oxygen Consumption	
Column B		Ts65Dn postnatal	
vs.		vs.	

Column A	WT postnatal
Mann Whitney test	
P value	0.0493
Exact or approximate P value?	Exact
P value summary	*
Significantly different (P < 0.05)?	Yes
One- or two-tailed P value?	Two-tailed
Sum of ranks in column A,B	88 , 188
Mann-Whitney U	33

Proton Leak

	WT	Ts65Dn
D'Agostino & Pearson normality test		
K2	0.5635	1.898
P value	0.7545	0.3872
Passed normality test (alpha=0.05)?	Yes	Yes
P value summary	ns	ns

Table Analyzed	Proton Leak
Column B	Ts65Dn postnatal
vs.	vs.
Column A	WT postnatal
Unpaired t test with Welch's correction	
P value	0.0050
P value summary	**
Significantly different (P < 0.05)?	Yes
One- or two-tailed P value?	Two-tailed
Welch-corrected t, df	t=3.257 df=15.95

ATP Production

	WT	Ts65Dn
D'Agostino & Pearson normality test		
K2	7.302	2.536
P value	0.0260	0.2813
Passed normality test (alpha=0.05)?	No	Yes
P value summary	*	ns

Table Analyzed	ATP Production
Column B	Ts65Dn postnatal
vs.	vs.
Column A	WT postnatal
Mann Whitney test	
P value	0.0121
Exact or approximate P value?	Exact
P value summary	*
Significantly different (P < 0.05)?	Yes
One- or two-tailed P value?	Two-tailed
Sum of ranks in column A,B	80 , 196
Mann-Whitney U	25

Maximal OCR

	WT	Ts65Dn
D'Agostino & Pearson normality test		
K2	3.515	3.047
P value	0.1724	0.2179
Passed normality test (alpha=0.05)?	Yes	Yes
P value summary	ns	ns

Table Analyzed	Maximal OCR
Column B	Ts65Dn postnatal
vs.	vs.
Column A	WT postnatal
Unpaired t test with Welch's correction	
P value	0.0114
P value summary	*
Significantly different (P < 0.05)?	Yes
One- or two-tailed P value?	Two-tailed
Welch-corrected t, df	t=2.819 df=18.03

Spare Capacity

	WT	Ts65Dn
D'Agostino & Pearson normality test		
K2	2.947	3.69
P value	0.2291	0.1581
Passed normality test (alpha=0.05)?	Yes	Yes
P value summary	ns	ns

Table Analyzed	Spare Capacity
Column B	Ts65Dn postnatal
vs.	vs.
Column A	WT postnatal
Unpaired t test with Welch's correction	
P value	0.0201
P value summary	*
Significantly different (P < 0.05)?	Yes
One- or two-tailed P value?	Two-tailed
Welch-corrected t, df	t=2.567 df=16.86

Coupling Efficiency

	WT	Ts65Dn
D'Agostino & Pearson normality test		
K2	0.8569	0.2618
P value	0.6515	0.8773
Passed normality test (alpha=0.05)?	Yes	Yes
P value summary	ns	ns

Table Analyzed	Coupling Efficiency
Column B	Ts65Dn postnatal
vs.	vs.
Column A	WT postnatal
Unpaired t test with Welch's correction	
P value	0.2421
P value summary	ns
Significantly different (P < 0.05)?	No
One- or two-tailed P value?	Two-tailed
Welch-corrected t, df	t=1.231 df=11.88

Data expressed as % of baseline values (Analysis done with GraphPad Prism)

non-mito OCR (% baseline)		Maximal OCR (% baseline)		Proton Leak (% baseline)		ATP Production (% baseline)		Spare OCR (% baseline)	
WT	Ts65Dn	WT	Ts65Dn	WT	Ts65Dn	WT	Ts65Dn	WT	Ts65Dn
26.9131	26.87772	222.9553	180.0022	16.76691	15.37445	83.23309	84.62555	222.7	179.94
34.5924	30.3227	214.3007	259.3862	16.80641	17.33562	83.19359	82.66439	214.32	260.58
34.45479	25.23355	209.6504	215.8588	22.74027	17.98597	77.25974	82.01404	209.49	215.47
30.86645	36.92455	199.282	259.1219	23.55037	18.7034	76.44963	81.29659	199.3	259.11
35.7722	29.23418	206.3868	252.7338	22.87955	19.98119	77.12046	80.01881	206.45	252.74
33.46315	29.00927	189.1123	246.529	11.21575	23.82736	88.78425	76.17264	189.53	246.52
33.17363	29.6596	178.8305	251.86	22.73438	20.35589	75.72126	79.64412	181.62	251.89
43.02762	25.26908	181.3385	172.0419	31.54966	15.84333	69.51266	84.15668	178.12	172.84
45.31118	26.10456	179.8102	179.3721	23.59041	17.46621	77.96622	82.53379	173.37	180.44
36.81379	27.35376	232.0611	127.3593	25.5928	21.45228	73.17887	76.05142	234.95	130.66
	28.89264		225.3829		19.83005		77.43182		231.77
	34.40977		231.8404		22.02143		76.41978		235.46
	30.29446		169.7263		20.29748		78.65015		172.09

Non-mito OCR (% baseline)

	WT	Ts65Dn
D'Agostino & Pearson normality test		
K2	1.082	4.191
P value	0.5823	0.1230
Passed normality test (alpha=0.05)?	Yes	Yes
P value summary	ns	ns

Table Analyzed	% Non-mitochondrial Oxygen Consumption
Column B	Ts65Dn postnatal
vs.	vs.

Column A	WT postnatal
Unpaired t test with Welch's correction	
P value	0.0062
P value summary	**
Significantly different (P < 0.05)?	Yes
One- or two-tailed P value?	Two-tailed
Welch-corrected t, df	t=3.203 df=14.36

Maximal OCR (% baseline)

	WT	Ts65Dn
D'Agostino & Pearson normality test		
K2	1.173	1.414
P value	0.5561	0.4930
Passed normality test (alpha=0.05)?	Yes	Yes
P value summary	ns	ns
Table Analyzed	% Maximal respiration	
Column B	Ts65Dn postnatal	
vs.	vs.	
Column A	WT postnatal	
Unpaired t test with Welch's correction		
P value	0.3877	
P value summary	ns	
Significantly different (P < 0.05)?	No	
One- or two-tailed P value?	Two-tailed	
Welch-corrected t, df	t=0.8861 df=17.32	

Proton Leak (% baseline)

	WT	Ts65Dn
D'Agostino & Pearson normality test		
K2	0.8223	0.07771
P value	0.6629	0.9619
Passed normality test (alpha=0.05)?	Yes	Yes
P value summary	ns	ns
Table Analyzed	% Proton Leak	
Column B	Ts65Dn postnatal	
vs.	vs.	
Column A	WT postnatal	
Unpaired t test with Welch's correction		
P value	0.2161	
P value summary	ns	
Significantly different (P < 0.05)?	No	
One- or two-tailed P value?	Two-tailed	
Welch-corrected t, df	t=1.308 df=11.67	

ATP Production (% baseline)

	WT	Ts65Dn
D'Agostino & Pearson normality test		
K2	0.7861	2.365
P value	0.6750	0.3064
Passed normality test (alpha=0.05)?	Yes	Yes
P value summary	ns	ns
Table Analyzed	% ATP Production	
Column B	Ts65Dn postnatal	
vs.	vs.	
Column A	WT postnatal	
Unpaired t test with Welch's correction		
P value	0.3473	
P value summary	ns	
Significantly different (P < 0.05)?	No	
One- or two-tailed P value?	Two-tailed	
Welch-corrected t, df	t=0.9748 df=13.1	

Spare OCR (% baseline)

	WT	Ts65Dn
D'Agostino & Pearson normality test		
K2	0.5981	1.597
P value	0.7415	0.4501

Passed normality test (alpha=0.05)?	Yes	Yes
P value summary	ns	ns
Table Analyzed	Spare Respiratory Capacity as a %	
Column B	Ts65Dn postnatal	
vs.	vs.	
Column A	WT postnatal	
Unpaired t test with Welch's correction		
P value	0.3243	
P value summary	ns	
Significantly different (P < 0.05)?	No	
One- or two-tailed P value?	Two-tailed	
Welch-corrected t, df	t=1.013 df=18.05	

Figure 3b (Data Analysis done with Statistica)

	C:\Users\Bruna\Desktop\Brain Sciences\Resultados para anova_nov21.xlsx : Pup-adult		
	Genotype	Basal	Oligomycin
1	WT	68.27	98.14
2	WT	21.90	37.36
3	WT	22.73	51.94
4	WT	22.15	38.62
5	WT	33.64	54.42
6	WT	27.49	56.74
7	WT	27.30	58.32
8	WT	30.04	54.28
9	WT	23.27	46.68
10	WT	18.35	44.54
11	Ts65Dn	25.98	54.75
12	Ts65Dn	96.15	165.41
13	Ts65Dn	72.04	120.71
14	Ts65Dn	65.46	108.13
15	Ts65Dn	36.08	69.08
16	Ts65Dn	29.09	60.95
17	Ts65Dn	30.15	56.00
18	Ts65Dn	18.07	37.02
19	Ts65Dn	37.00	70.28
20	Ts65Dn	30.92	62.04
21	Ts65Dn	28.21	66.35
22	Ts65Dn	28.71	62.99
23	Ts65Dn	25.49	63.93

	Repeated Measures Analysis of Variance (Stats dez_2021-acc2) Sigma-restricted parameterization Effective hypothesis decomposition				
Effect	SS	Degr. of Freedom	MS	F	p
Intercept	113742.3	1	113742.3	99.3736	0.000000
Genotype	3149.1	1	3149.1	2.7513	0.112038
Error	24036.4	21	1144.6		
TREATM	10541.3	1	10541.3	210.3052	0.000000

TREATM*Genotype	399.8	1	399.8	7.9760	0.010163
Error	1052.6	21	50.1		

Cell No.	LSD test; variable DV_1 (Stats dez_2021-acc2) Probabilities for Post Hoc Tests Error: Between; Within; Pooled MS = 597.36, df = 22.836					
	Genotype	TREATM	{1} 29.514	{2} 54.104	{3} 40.258	{4} 76.742
1	WT	Basal		0.000000	0.306929	0.000130
2	WT	Oligomycin	**0.000000**		0.191239	**0.038037**
3	Ts65Dn	Basal	0.306929	0.191239		**0.000000**
4	Ts65Dn	Oligomycin	0.000130	0.038037	0.000000	

Figures 3c-e (Data Analysis done with GraphPad Prism)

Figure 3c

Glycolytic capacity

D'Agostino & Pearson normality test		
K2	1.301	11
P value	0.5219	0.0041
Passed normality test (alpha=0.05)?	Yes	No
P value summary	ns	**
Table Analyzed	Glycolytic capacity pup	
Column B	Ts65Dn neonatal	
vs.	vs.	
Column A	WT neonatal	
Mann Whitney test		
P value	0.0020	
Exact or approximate P value?	Exact	
P value summary	**	
Significantly different (P < 0.05)?	Yes	
One- or two-tailed P value?	Two-tailed	
Sum of ranks in column A,B	72 , 204	
Mann-Whitney U	17	

Figure 3d

Glycolytic reserve

D'Agostino & Pearson normality test		
K2	0.3955	0.5715
P value	0.8206	0.7514
Passed normality test (alpha=0.05)?	Yes	Yes
P value summary	ns	ns
Table Analyzed	Glycolytic reserve	
Column B	Ts65Dn neonatal	
vs.	vs.	
Column A	WT neonatal	
Unpaired t test with Welch's correction		
P value	0.5225	
P value summary	ns	
Significantly different (P < 0.05)?	No	
One- or two-tailed P value?	Two-tailed	
Welch-corrected t, df	t=0.6528 df=17.23	

Figure 3e

OCR/ECAR

D'Agostino & Pearson normality test		
K2	2.797	9.367
P value	0.2470	0.0092
Passed normality test (alpha=0.05)?	Yes	No
P value summary	ns	**
Table Analyzed	OCR/ECAR	
Column B	Ts65Dn neonatal	
vs.	vs.	
Column A	WT neonatal	
Mann Whitney test		
P value	0.2569	
Exact or approximate P value?	Exact	
P value summary	ns	
Significantly different (P < 0.05)?	No	
One- or two-tailed P value?	Two-tailed	
Sum of ranks in column A,B	101 , 175	
Mann-Whitney U	46	

Figure 4 (Data Analysis done with Statistica)

Figure 4b (One-way ANOVA)

	Fig4b-BasalOCR-Data	
	Genotype-Treatm	Oxygen Consump
1	WT-Basal	115.93
2	WT-Basal	63.35
3	WT-Basal	60.47
4	WT-Basal	74.17
5	WT-Basal	62.38
6	WT-Basal	132.24
7	WT-Basal	74.02
8	WT-Basal	54.65
9	WT-Basal	47.07
10	WT-Basal	65.55
11	TS-Basal	213.94
12	TS-Basal	197.29
13	TS-Basal	210.49
14	TS-Basal	82.18
15	TS-Basal	120.57
16	TS-Basal	99.67
17	TS-Basal	95.71
18	TS-Basal	133.44
19	TS-Basal	111.45
20	TS-Basal	101.19
21	TS-Basal	73.33
22	TS-Basal	60.14
23	TS-Basal	92.79
24	WT-Glut	83.23
25	WT-Glut	76.41
26	WT-Glut	191.69
27	WT-Glut	152.63

28	WT-Glut	78.46
29	WT-Glut	43.87
30	WT-Glut	62.77
31	WT-Glut	48
32	WT-Glut	73.3
33	TS-Glut	164.79
34	TS-Glut	75.1
35	TS-Glut	138
36	TS-Glut	204.97
37	TS-Glut	101.92
38	TS-Glut	88.19
39	TS-Glut	101.19
40	TS-Glut	60.72
41	TS-Glut	90.63

Effect	Univariate Tests of Significance for Oxygen Consump (Stats dez_2021-Fig4b-BasalOCR) Sigma-restricted parameterization Effective hypothesis decomposition				
	SS	Degr. of Freedom	MS	F	p
Intercept	403757.4	1	403757.4	196.4673	0.000000
Genotype -Treatm	15321.0	3	5107.0	2.4850	0.075783
Error	76038.2	37	2055.1		

	Fig4b- Non-MitoOCR -Data	
	Genotype-Treatm	Oxygen Consump
1	WT-Basal	31.2
2	WT-Basal	21.91
3	WT-Basal	20.83
4	WT-Basal	22.89
5	WT-Basal	22.32
6	WT-Basal	44.25
7	WT-Basal	24.56
8	WT-Basal	23.51
9	WT-Basal	21.33
10	WT-Basal	24.13
11	TS-Basal	57.5
12	TS-Basal	59.83
13	TS-Basal	53.11
14	TS-Basal	30.35
15	TS-Basal	35.25
16	TS-Basal	28.91
17	TS-Basal	28.39
18	TS-Basal	33.72
19	TS-Basal	29.09
20	TS-Basal	27.68
21	TS-Basal	21.19
22	TS-Basal	20.69

23	TS-Basal	28.11
24	WT-Glut	32.24
25	WT-Glut	25.84
26	WT-Glut	51.46
27	WT-Glut	49.84
28	WT-Glut	27.06
29	WT-Glut	21.05
30	WT-Glut	23.04
31	WT-Glut	21.69
32	WT-Glut	21.57
33	TS-Glut	63.02
34	TS-Glut	20.45
35	TS-Glut	32.26
36	TS-Glut	55.06
37	TS-Glut	30.31
38	TS-Glut	29.03
39	TS-Glut	28.48
40	TS-Glut	21.44
41	TS-Glut	23.06

Effect	Univariate Tests of Significance for Oxygen Consump (Stats dez_2021-Fig4b-Non-MitoOCR) Sigma-restricted parameterization Effective hypothesis decomposition				
	SS	Degr. of Freedom	MS	F	p
Intercept	38959.86	1	38959.86	261.3601	0.000000
Genotype -Treatm	541.15	3	180.38	1.2101	0.319602
Error	5515.44	37	149.07		

	**Fig4b- Proton Leak -Data**	
	Genotype-Treatm	Oxygen Consump
1	WT-Basal	19.44
2	WT-Basal	10.65
3	WT-Basal	13.75
4	WT-Basal	17.47
5	WT-Basal	14.27
6	WT-Basal	14.83
7	WT-Basal	16.83
8	WT-Basal	17.24
9	WT-Basal	11.1
10	WT-Basal	16.78
11	TS-Basal	32.89
12	TS-Basal	34.2
13	TS-Basal	37.86
14	TS-Basal	15.37
15	TS-Basal	24.09
16	TS-Basal	23.75
17	TS-Basal	19.48

18	TS-Basal	21.14
19	TS-Basal	19.47
20	TS-Basal	21.71
21	TS-Basal	14.54
22	TS-Basal	13.24
23	TS-Basal	18.83
24	WT-Glut	16.5
25	WT-Glut	18.32
26	WT-Glut	38.85
27	WT-Glut	28.34
28	WT-Glut	20.64
29	WT-Glut	11.39
30	WT-Glut	17.78
31	WT-Glut	15.54
32	WT-Glut	17.85
33	TS-Glut	27.04
34	TS-Glut	18.45
35	TS-Glut	28.08
36	TS-Glut	37.34
37	TS-Glut	26.13
38	TS-Glut	23.01
39	TS-Glut	26.84
40	TS-Glut	15.4
41	TS-Glut	20.28

	Univariate Tests of Significance for Oxygen Consump (Stats dez_2021-Fig4b-Proton-Leak) Sigma-restricted parameterization Effective hypothesis decomposition				
Effect	SS	Degr. of Freedom	MS	F	p
Intercept	17408.57	1	17408.57	387.3278	0.000000
Genotype -Treatm	501.33	3	167.11		0.019644
Error	1662.98	37	44.95		

	LSD test; variable Oxygen Consump (Stats dez_2021-Fig4b-Proton-Leak) Probabilities for Post Hoc Tests Error: Between MS = 44.945, df = 37.000				
Cell No.	Genotype-Treatm	{1} 15.236	{2} 22.813	{3} 20.579	{4} 24.730
1	WT-Basal		0.010737	0.091152	0.003869
2	TS-Basal	0.010737		0.447052	0.513727
3	WT-Glut	0.091152	0.447052		**0.197103**
4	TS-Glut	0.003869	0.513727	0.197103	

	Fig4b- ATP Production -Data	
	Genotype-Treatm	Oxygen Consump
1	WT-Basal	96.49
2	WT-Basal	52.7
3	WT-Basal	46.72
4	WT-Basal	56.7
5	WT-Basal	48.11
6	WT-Basal	117.41
7	WT-Basal	56.05
8	WT-Basal	37.99
9	WT-Basal	36.7
10	WT-Basal	47.97
11	TS-Basal	181.05
12	TS-Basal	163.09
13	TS-Basal	172.63
14	TS-Basal	66.81
15	TS-Basal	96.48
16	TS-Basal	75.92
17	TS-Basal	76.23
18	TS-Basal	112.3
19	TS-Basal	91.99
20	TS-Basal	76.96
21	TS-Basal	56.78
22	TS-Basal	45.96
23	TS-Basal	72.98
24	WT-Glut	90.95
25	WT-Glut	60.81
26	WT-Glut	157.08
27	WT-Glut	129.19
28	WT-Glut	76.8
29	WT-Glut	45.31
30	WT-Glut	65.6
31	WT-Glut	36.18
32	WT-Glut	58.83
33	TS-Glut	152.94
34	TS-Glut	78.48
35	TS-Glut	122.86
36	TS-Glut	184.45
37	TS-Glut	94.85
38	TS-Glut	90.05
39	TS-Glut	88.3
40	TS-Glut	62.04
41	TS-Glut	80.63

Effect	Univariate Tests of Significance for Oxygen Consump (Stats dez_2021-Fig4b-ATP-Production) Sigma-restricted parameterization Effective hypothesis decomposition				
	SS	Degr. of Freedom	MS	F	p
Intercept	298202.6	1	298202.6	197.5879	0.000000
Genotype-Treatm	13088.0	3	4362.7	2.8907	0.048269

Error	55840.9	37	1509.2		

Cell No.	LSD test; variable Oxygen Consump (Stats dez_2021-Fig4b-ATP-Production) Probabilities for Post Hoc Tests Error: Between MS = 1509.2, df = 37.000				
	Genotype-Treatm	{1} 59.684	{2} 99.168	{3} 80.083	{4} 106.07
1	WT-Basal		0.020730	0.260447	0.013366
2	TS-Basal	**0.020730**		0.264553	0.684509
3	WT-Glut	0.260447	0.264553		**0.164322**
4	TS-Glut	0.013366	0.684509	0.164322	

	**Fig4b- MaxOCR -Data**	
	Genotype-Treatm	Oxygen Consump
1	WT-Basal	258.46
2	WT-Basal	135.76
3	WT-Basal	126.78
4	WT-Basal	147.8
5	WT-Basal	128.75
6	WT-Basal	250.09
7	WT-Basal	132.37
8	WT-Basal	99.1
9	WT-Basal	84.63
10	WT-Basal	152.11
11	TS-Basal	385.1
12	TS-Basal	511.76
13	TS-Basal	454.36
14	TS-Basal	212.95
15	TS-Basal	304.73
16	TS-Basal	245.71
17	TS-Basal	241.06
18	TS-Basal	229.57
19	TS-Basal	199.91
20	TS-Basal	128.88
21	TS-Basal	165.28
22	TS-Basal	139.42
23	TS-Basal	157.49
24	WT-Glut	196.68
25	WT-Glut	190.8
26	WT-Glut	420.76
27	WT-Glut	281.14
28	WT-Glut	212.7
29	WT-Glut	82.87
30	WT-Glut	152.91
31	WT-Glut	96.17
32	WT-Glut	132.92
33	TS-Glut	317.27
34	TS-Glut	211.34

35	TS-Glut	379.85
36	TS-Glut	483.5
37	TS-Glut	345.9
38	TS-Glut	246.45
39	TS-Glut	221.47
40	TS-Glut	167.22
41	TS-Glut	248.93

Effect	Univariate Tests of Significance for Oxygen Consump (Stats dez_2021-Fig4b-MaxOCR) Sigma-restricted parameterization Effective hypothesis decomposition				
	SS	Degr. of Freedom	MS	F	p
Intercept	2024593	1	2024593	200.8041	0.000000
Genotype-Treatm	116473	3	38824	3.8507	0.017058
Error	373050	37	10082		

Cell No.	LSD test; variable Oxygen Consump (Stats dez_2021-Fig4b-MaxOCR) Probabilities for Post Hoc Tests Error: Between MS = 10082., df = 37.000				
	Genotype-Treatm	{1} 151.59	{2} 259.71	{3} 196.33	{4} 291.33
1	WT-Basal		0.014683	0.338441	0.004457
2	TS-Basal	**0.014683**		0.153919	0.472332
3	WT-Glut	0.338441	0.153919		**0.052103**
4	TS-Glut	0.004457	0.472332	0.052103	

	**Fig4b- Spare Capacity -Data**	
	Genotype-Treatm	Oxygen Consump
1	WT-Basal	142.54
2	WT-Basal	72.41
3	WT-Basal	66.31
4	WT-Basal	73.63
5	WT-Basal	66.37
6	WT-Basal	117.84
7	WT-Basal	59.49
8	WT-Basal	43.87
9	WT-Basal	36.83
10	WT-Basal	87.37
11	TS-Basal	171.16
12	TS-Basal	314.46
13	TS-Basal	243.87
14	TS-Basal	130.77
15	TS-Basal	184.16
16	TS-Basal	146.04
17	TS-Basal	145.35

18	TS-Basal	96.13
19	TS-Basal	88.46
20	TS-Basal	30.21
21	TS-Basal	93.95
22	TS-Basal	80.22
23	TS-Basal	65.68
24	WT-Glut	89.23
25	WT-Glut	111.66
26	WT-Glut	224.84
27	WT-Glut	123.61
28	WT-Glut	115.26
29	WT-Glut	26.17
30	WT-Glut	69.54
31	WT-Glut	44.45
32	WT-Glut	56.24
33	TS-Glut	137.28
34	TS-Glut	114.42
35	TS-Glut	228.92
36	TS-Glut	261.71
37	TS-Glut	224.92
38	TS-Glut	133.38
39	TS-Glut	106.33
40	TS-Glut	89.77
41	TS-Glut	148.02

Effect	Univariate Tests of Significance for Oxygen Consump (Stats dez_2021-Fig4b-Spare-Capacity) Sigma-restricted parameterization Effective hypothesis decomposition				
	SS	Degr. of Freedom	MS	F	p
Intercept	554818.5	1	554818.5	146.8061	0.000000
Genotype -Treatm	42895.8	3	14298.6	3.7834	0.018323
Error	139832.6	37	3779.3		

Cell No.	LSD test; variable Oxygen Consump (Stats dez_2021-Fig4b-Spare-Capacity) Probabilities for Post Hoc Tests Error: Between MS = 3779.3, df = 37.000				
	Genotype-Treatm	{1} 76.666	{2} 137.73	{3} 95.667	{4} 160.53
1	WT-Basal		0.023581	0.505329	0.005218
2	TS-Basal	**0.023581**		0.123119	0.397894
3	WT-Glut	0.505329	0.123119		**0.031315**
4	TS-Glut	0.005218	0.397894	0.031315	

	Fig4b- Coupling Efficiency -Data	
	Genotype-Treatm	Oxygen Consump
1	WT-Basal	83.23
2	WT-Basal	83.18
3	WT-Basal	77.24
4	WT-Basal	76.33
5	WT-Basal	77.12
6	WT-Basal	88.82
7	WT-Basal	76.9
8	WT-Basal	68.77
9	WT-Basal	76.93
10	WT-Basal	74.1
11	TS-Basal	84.64
12	TS-Basal	82.56
13	TS-Basal	82.01
14	TS-Basal	81.3
15	TS-Basal	80.04
16	TS-Basal	76.17
17	TS-Basal	79.64
18	TS-Basal	84.15
19	TS-Basal	82.39
20	TS-Basal	77.99
21	TS-Basal	79.62
22	TS-Basal	77.67
23	TS-Basal	79.49
24	WT-Glut	84.64
25	WT-Glut	76.87
26	WT-Glut	80.15
27	WT-Glut	82.02
28	WT-Glut	78.83
29	WT-Glut	79.9
30	WT-Glut	78.7
31	WT-Glut	69.9
32	WT-Glut	76.73
33	TS-Glut	85.26
34	TS-Glut	80.96
35	TS-Glut	81.4
36	TS-Glut	83.16
37	TS-Glut	78.42
38	TS-Glut	79.62
39	TS-Glut	76.68
40	TS-Glut	80.11
41	TS-Glut	79.92

Effect	Univariate Tests of Significance for Oxygen Consump (Stats dez_2021-Fig4b-Coupling-Efficiency) Sigma-restricted parameterization Effective hypothesis decomposition				
	SS	Degr. of Freedom	MS	F	p
Intercept	253517.4	1	253517.4	17380.77	0.000000
Genotype-Treatm	48.2	3	16.1	1.10	0.360419

Error	539.7	37	14.6		

Figure 4d (RM ANOVA)

	Fig4d- Basal and oligomycin ECAR in Astrocytes treated with Glutamate -Data		
	Genotype-Treatm	Basal	Oligomycin
1	WT-Basal	68.27	98.14
2	WT-Basal	21.90	37.36
3	WT-Basal	22.73	51.94
4	WT-Basal	22.15	38.62
5	WT-Basal	33.64	54.42
6	WT-Basal	27.49	56.74
7	WT-Basal	27.30	58.32
8	WT-Basal	30.04	54.28
9	WT-Basal	23.27	46.68
10	WT-Basal	18.35	44.54
11	Ts65Dn-Basal	25.98	54.75
12	Ts65Dn-Basal	96.15	165.41
13	Ts65Dn-Basal	72.04	120.71
14	Ts65Dn-Basal	65.46	108.13
15	Ts65Dn-Basal	36.08	69.08
16	Ts65Dn-Basal	29.09	60.95
17	Ts65Dn-Basal	30.15	56.00
18	Ts65Dn-Basal	18.07	37.02
19	Ts65Dn-Basal	37.00	70.28
20	Ts65Dn-Basal	30.92	62.04
21	Ts65Dn-Basal	28.21	66.35
22	Ts65Dn-Basal	28.71	62.99
23	Ts65Dn-Basal	25.49	63.93
24	WT-Glut	29.62	52.90
25	WT-Glut	40.51	81.16
26	WT-Glut	42.50	78.55
27	WT-Glut	26.59	54.55
28	WT-Glut	22.36	41.62
29	WT-Glut	16.92	46.10
30	WT-Glut	20.81	37.12
31	WT-Glut	30.26	52.21
32	WT-Glut	54.86	91.79
33	Ts65Dn-Glut	23.86	48.36
34	Ts65Dn-Glut	51.87	93.58
35	Ts65Dn-Glut	59.92	100.72
36	Ts65Dn-Glut	26.52	61.60
37	Ts65Dn-Glut	26.30	55.86
38	Ts65Dn-Glut	36.34	54.51
39	Ts65Dn-Glut	16.20	38.84
40	Ts65Dn-Glut	66.71	114.79
41	Ts65Dn-Glut	28.49	50.25

Effect	Repeated Measures Analysis of Variance (Stats dez_2021-acc-Glut-Oligomycin) Sigma-restricted parameterization Effective hypothesis decomposition				
	SS	Degr. of Freedom	MS	F	p
Intercept	198285.6	1	198285.6	204.6393	0.000000
Genotype-Glut	3725.5	3	1241.8	1.2816	0.294956
Error	35851.2	37	969.0		
Treatment	18156.8	1	18156.8	376.8680	0.000000
Treament*Genotype-Glut	439.8	3	146.6	3.0430	0.040815
Error	1782.6	37	48.2		

Cell No.	LSD test; variable DV_1 (Stats dez_2021-acc-Glut-Oligomycin) Probabilities for Post Hoc Tests Error: Between; Within; Pooled MS = 508.56, df = 40.670									
	Genotype-Glut	Treatment	{1} 29.514	{2} 54.104	{3} 40.258	{4} 76.742	{5} 31.603	{6} 59.556	{7} 37.357	{8} 68.723
1	WT-no-Glut	Basal		**0.000000**	**0.264003**	0.000012	0.841203	0.006004	0.453476	0.000498
2	WT-no-Glut	Oligomycin	0.000000		0.152050	**0.021746**	0.035779	0.601657	0.113765	0.165875
3	Ts65Dn-no-Glut	Basal	0.264003	0.152050		**0.000000**	0.381359	0.055270	0.768235	0.005823
4	Ts65Dn-no-Glut	Oligomycin	0.000012	0.021746	0.000000		**0.000039**	0.086367	0.000240	0.417026
5	WT-Glut	Basal	0.841203	0.035779	0.381359	0.000039		**0.000000**	**0.591327**	0.001171
6	WT-Glut	Oligomycin	0.006004	0.601657	0.055270	0.086367	0.000000		**0.043087**	**0.393537**
7	Ts65Dn-Glut	Basal	0.453476	0.113765	0.768235	0.000240	0.591327	0.043087		**0.000000**
8	Ts65Dn-Glut	Oligomycin	0.000498	0.165875	0.005823	0.417026	0.001171	0.393537	0.000000	

Figure 4e (RM ANOVA)

	Fig4e- glycolytic capacity in Astrocytes treated with Glutamate -Data	
	Genotype-Treatm	Oxygen Consump
1	WT-Basal	29.87
2	WT-Basal	15.46
3	WT-Basal	29.21
4	WT-Basal	16.47
5	WT-Basal	20.78
6	WT-Basal	29.24
7	WT-Basal	31.02
8	WT-Basal	24.24
9	WT-Basal	23.4
10	WT-Basal	26.19
11	TS-Basal	28.76
12	TS-Basal	69.27

13	TS-Basal	48.67
14	TS-Basal	42.67
15	TS-Basal	33.01
16	TS-Basal	31.86
17	TS-Basal	25.85
18	TS-Basal	18.96
19	TS-Basal	33.28
20	TS-Basal	31.11
21	TS-Basal	38.13
22	TS-Basal	34.27
23	TS-Basal	38.44
24	WT-Glut	23.28
25	WT-Glut	40.65
26	WT-Glut	36.05
27	WT-Glut	27.97
28	WT-Glut	19.26
29	WT-Glut	29.18
30	WT-Glut	16.31
31	WT-Glut	21.95
32	WT-Glut	36.93
33	TS-Glut	24.5
34	TS-Glut	41.71
35	TS-Glut	40.8
36	TS-Glut	35.08
37	TS-Glut	29.57
38	TS-Glut	18.17
39	TS-Glut	22.64
40	TS-Glut	48.08
41	TS-Glut	21.75

Effect	Univariate Tests of Significance for Oxygen Consump (Stats dez_2021-Fig4e- glycolytic capacity) Sigma-restricted parameterization Effective hypothesis decomposition				
	SS	Degr. of Freedom	MS	F	p
Intercept	36312.62	1	36312.62	376.7947	0.000000
Genotype -Treatm	879.71	3	293.24	3.0427	0.040827
Error	3565.78	37	96.37		

Cell No.	LSD test; variable Oxygen Consump (Stats dez_2021-Fig4e-- glycolytic capacity) Probabilities for Post Hoc Tests Error: Between MS = 96.372, df = 37.000				
	Genotype-Treatm	{1} 24.588	{2} 36.483	{3} 27.953	{4} 31.367
1	WT-Basal		0.006565	0.460322	0.141369
2	TS-Basal	**0.006565**		0.052461	0.237036
3	WT-Glut	0.460322	0.052461		**0.465422**
4	TS-Glut	0.141369	0.237036	0.465422	

Figure 4f (One-way ANOVA)

	Fig4f- glycolytic reserve in Astrocytes treated with Glutamate -Data	
	Genotype-Treatm	Oxygen Consump
1	WT-Basal	0.4375275
2	WT-Basal	0.7059361
3	WT-Basal	1.285086
4	WT-Basal	0.7435666
5	WT-Basal	0.617717
6	WT-Basal	1.06366
7	WT-Basal	1.136264
8	WT-Basal	0.8069241
9	WT-Basal	1.005587
10	WT-Basal	1.427248
11	TS-Basal	1.107005
12	TS-Basal	0.7204368
13	TS-Basal	0.6755969
14	TS-Basal	0.6518484
15	TS-Basal	0.9149113
16	TS-Basal	1.095222
17	TS-Basal	0.8573798
18	TS-Basal	1.049253
19	TS-Basal	0.8994595
20	TS-Basal	1.006145
21	TS-Basal	1.351648
22	TS-Basal	1.193661
23	TS-Basal	1.508042
24	WT-Glut	0.9756916
25	WT-Glut	0.78369
26	WT-Glut	0.6016355
27	WT-Glut	1.054676
28	WT-Glut	0.7323194
29	WT-Glut	0.802972
30	WT-Glut	1.00679
31	WT-Glut	0.3290361
32	WT-Glut	1.296244
33	TS-Glut	1.026823
34	TS-Glut	0.8041257
35	TS-Glut	0.6809079
36	TS-Glut	1.322775
37	TS-Glut	1.124335
38	TS-Glut	0.5
39	TS-Glut	1.397531
40	TS-Glut	0.7207315
41	TS-Glut	0.7634258

Effect	Univariate Tests of Significance for Oxygen Consump (Stats dez_2021-Fig4e-glycolytic reserve) Sigma-restricted parameterization Effective hypothesis decomposition				
	SS	Degr. of Freedom	MS	F	p

Intercept	34.19838	1	34.19838	414.5550	0.000000
Genotype -Treatm	0.13739	3	0.04580	0.5551	0.647944
Error	3.05228	37	0.08249		

Figure 4g (One-way ANOVA)

	Fig4g- OCR/ECAR ratio in Astrocytes treated with Glutamate -Data	
	Genotype-Treatm	Oxygen Consump
1	WT-Basal	1.69811
2	WT-Basal	2.892694
3	WT-Basal	2.660361
4	WT-Basal	3.348533
5	WT-Basal	1.85434
6	WT-Basal	4.810476
7	WT-Basal	2.711355
8	WT-Basal	1.819241
9	WT-Basal	2.022776
10	WT-Basal	3.572207
11	TS-Basal	8.234796
12	TS-Basal	2.051898
13	TS-Basal	2.921849
14	TS-Basal	1.255423
15	TS-Basal	3.341741
16	TS-Basal	3.426263
17	TS-Basal	3.174461
18	TS-Basal	7.384615
19	TS-Basal	3.012162
20	TS-Basal	3.272639
21	TS-Basal	2.599433
22	TS-Basal	2.09474
23	TS-Basal	3.640251
24	WT-Glut	3.488265
25	WT-Glut	1.473106
26	WT-Glut	3.199099
27	WT-Glut	5.755279
28	WT-Glut	2.98327
29	WT-Glut	1.20721
30	WT-Glut	3.874691
31	WT-Glut	0.7195323
32	WT-Glut	2.572833
33	TS-Glut	6.906538
34	TS-Glut	1.44785
35	TS-Glut	2.303071
36	TS-Glut	7.728884
37	TS-Glut	3.875285
38	TS-Glut	2.426802
39	TS-Glut	6.246296
40	TS-Glut	0.9102083
41	TS-Glut	3.181116

Effect	Univariate Tests of Significance for Oxygen Consump (Stats dez_2021-Fig4g- OCR/ECAR ratio) Sigma-restricted parameterization Effective hypothesis decomposition				
	SS	Degr. of Freedom	MS	F	p
Intercept	423.9883	1	423.9883	125.1160	0.000000
Genotype -Treatm	9.3890	3	3.1297	0.9235	0.438991
Error	125.3842	37	3.3888		

Figure 5 (RM-ANOVA done with Statistica)

	Genotype	150	300	450	600	750	900	1050
1	Ct	97.61759	96.4434	95.46321	92.81193	94.2618	97.86264	89.0273
2	Ct	97.92319	92.12501	94.00805	98.74298	98.93675	96.51711	62.19009
3	Ct	102.0518	100.2863	108.3347	110.36	98.0807	100.2227	20.25343
4	Ct	124.4006	117.3762	111.9468	93.66219	60.64223	64.01183	41.71332
5	Ct	116.8843	116.7801	128.2759	115.8582	42.46049	35.28863	25.48288
6	Ct	102.9437	104.1087	107.266	108.0544	101.8641	72.84272	33.46408
7	Ct	117.9979	118.1127	115.4091	117.9184	96.65135	102.2177	35.54515
8	Ct	101.4795	93.75957	97.19947	93.0568	96.68164	82.17702	
9	Ct	103.2431	109.286	98.28512	98.67009	104.1181	65.71395	
10	Ct	81.26357	85.24964	82.858	83.46807	85.45924	79.92739	
11	Ct	101.2239	103.1144	104.046	102.9741	98.20698	83.36387	
12	Ct	94.71782	98.74387	95.9489	99.61708	99.1089	80.29202	
13	Ts	93.73476	89.94145	88.20133	86.31485	89.25842	79.6227	26.27256
14	Ts	94.52034	90.4986	94.50504	99.43749	96.89664	84.45644	32.93537
15	Ts	121.0847	115.8083	118.7408	112.1085	89.96216	93.53584	31.91087
16	Ts	105.8081	111.1565	113.0098	111.3973	107.3258	108.88	24.44363
17	Ts	105.8703	110.0295	104.8542	106.6442	85.95346	91.07613	55.48068
18	Ts	96.36415	91.83051	97.57475	106.5892	101.0806	75.49155	35.39568
19	Ts	102.8017	99.82188	105.6419	108.6218	105.5745	93.94888	

Effect	Repeated Measures Analysis of Variance (Cytotoxicity) Sigma-restricted parameterization Effective hypothesis decomposition				
	SS	Degr. of Freedom	MS	F	p
Intercept	742413.3	1	742413.3	2465.543	0.000000
Genotype	42.7	1	42.7	0.142	0.713520
Error	3312.3	11	301.1		
H2O2-Conc	45324.9	6	7554.2	35.196	0.000000
H2O2-Conc*Genotype	1084.9	6	180.8	0.842	0.541783
Error	14165.8	66	214.6		
